# Relationship Between Pain Alleviation and Disease-specific Health-related Quality of Life Measures in Patients With Chronic Low Back Pain Receiving Duloxetine: Exploratory Post Hoc Analysis of a Japanese Phase 3 Randomized Study

**DOI:** 10.5435/JAAOSGlobal-D-18-00086

**Published:** 2019-11-27

**Authors:** Hiroyuki Enomoto, Nao Sasaki, Shinji Fujikoshi, Aki Yoshikawa, Toshinaga Tsuji, Katsushi Takeshita

**Affiliations:** From the Medicines Development Unit Japan, Eli Lilly Japan K.K., Akasaka, Minato-Ku, Tokyo, Japan (Dr. Enomoto); the Medicines Development Unit Japan, Eli Lilly Japan K.K., Chuo-Ku, Kobe, Japan (Ms. Sasaki, Mr. Fujikoshi, and Ms. Yoshikawa); the Medical Affairs Department, Shionogi & Co., Ltd., Kita-ku, Osaka, Japan (Dr. Tsuji); and the Department of Orthopaedic Surgery, Jichi Medical University, Tochigi, Japan (Dr. Takeshita).

## Abstract

This post hoc analysis of a Japanese phase 3 randomized study (ClinicalTrials.gov identifier: NCT01855919) investigated relationships between pain severity (assessed by the Brief Pain Inventory [BPI]) and disease-specific health-related quality of life (assessed by the 24-item Roland-Morris Disability Questionnaire [RDQ-24]) in duloxetine-treated patients with chronic low back pain (CLBP).

**Methods:**

Patients with CLBP duration >6 months and BPI average score ≥4 received duloxetine 60 mg/d (N = 230) or placebo (N = 226) for 14 weeks. Spearman rank correlation coefficients were calculated for (1) BPI change from baseline and RDQ item change from baseline and (2) BPI change from baseline and the RDQ item baseline score in duloxetine-treated patients.

**Results:**

Duloxetine treatment significantly improved the RDQ-24 total score compared with placebo; the greatest improvements were observed for RDQ02, RDQ17, and RDQ13. The strongest correlations between BPI change from baseline and RDQ item change from baseline were for RDQ13, RDQ23, and RDQ10. The correlation coefficients for the correlations between BPI change from baseline and the RDQ item baseline score were generally small.

**Discussion:**

This post hoc analysis suggested that improvement in pain severity was associated with improvement in the RDQ-24 total score and in some individual RDQ items in duloxetine-treated patients with CLBP. Furthermore, positive responses to duloxetine in terms of the RDQ13, RDQ23, and RDQ10 items may correlate with better pain responses.

**Clinical Trial Registry:**

The study described in this manuscript was registered at www.clinicaltrials.gov (NCT01855919).

Patients with chronic low back pain (CLBP; usually defined as pain persisting for more than 3 months)^1,2^ experience high levels of disability, loss of work productivity, anxiety, depression due to chronic pain, and deterioration in health-related quality of life (HRQoL).^3,4,5,6,7^ As CLBP is a complex condition and up to 85% of patients with CLBP have no specific underlying causes,^8^ patients with CLBP are a heterogeneous cohort, with varying demographics, manifestations of pain, and radiological findings, and a comprehensive and multidisciplinary approach is required to determine the appropriate interventions.^9-11^ In addition, the potential for negative effects of treatment needs to be taken into consideration, such as the epidemic of addictions and overdose deaths associated with opioid use. Pharmacological treatments for CLBP recommended by international and Japanese treatment guidelines include NSAIDs, acetaminophen, muscle relaxants, antidepressants, and opioids.^1,10,12^ Gabapentin had been reported to be effective for pain relief (for radiculopathy)^12^; however, the recent literature suggests that there is limited evidence to support the use of gabapentinoids (gabapentin and pregabalin) in the treatment of CLBP.^13-15^ Duloxetine, a serotonin-norepinephrine reuptake inhibitor, has been shown to have analgesic efficacy in CLBP.^16,17,18,19^ The exact mechanism of action of duloxetine in inhibiting pain is unknown. However, by inhibiting the reuptake of serotonin and norepinephrine in the dorsal horn of the spinal cord, it is thought that duloxetine activates descending pain inhibitory pathways, thereby producing an analgesic effect.^19,20^ Preclinical studies have shown duloxetine to exhibit analgesic effects at the peripheral level as well, by modifying sodium channels^21^ and inhibiting P2X4 receptor function.^22^ Owing to its predominantly centrally acting analgesic effect, duloxetine may be effective against the central sensitization component of CLBP.^9,23^

The efficacy and safety of duloxetine have been demonstrated in Japanese patients with CLBP responding inadequately to NSAIDs in a randomized phase 3 study.^19^ Patients in the study had no radiculopathy symptoms, no other specific low back diseases, and no history of low back surgery and were diagnosed with CLBP as defined by Japanese^1^ and international^24^ guidelines. Duloxetine treatment significantly improved pain compared with placebo, as assessed by the Brief Pain Inventory (BPI) average pain severity score. In addition, duloxetine treatment significantly improved disease-specific HRQoL compared with placebo, as assessed by the 24-item Roland-Morris Disability Questionnaire (RDQ-24). The RDQ-24 is one of the most widely used functional scales for LBP and has a validated Japanese version.^25-27^ In this study, duloxetine treatment also significantly improved HRQoL compared with placebo, as assessed by the “General Health” and “Mental Health” subscales of the 36-Item Short-Form Health Survey and the “Work time missed” subscale of the Work Productivity and Activity Impairment Questionnaire, but not the European Quality of Life 5-Dimensions questionnaire.^19^ Treatment goals for CLBP should include improving HRQoL by reducing pain intensity. However, there is limited literature available evaluating the correlation between improvement in pain severity and HRQoL in patients with CLBP receiving analgesics.

The aim of the current post hoc analysis was first to investigate which RDQ item scores improved in parallel with the reduction in pain intensity, as assessed by the BPI, in duloxetine-treated patients in the previously conducted phase 3 study of duloxetine in Japanese patients with CLBP.^19^ Second, we assessed which RDQ item baseline scores correlated with the reduction in pain intensity to investigate which patient cohorts might preferentially respond to duloxetine in the treatment of CLBP.

## Methods

### Study Design

Full details of the study design have been published elsewhere.^19^ This was a multicenter, randomized, placebo-controlled, double-blind, phase 3 study conducted at 58 medical institutions in Japan from May 2013 to July 2014. The study protocol was approved by the institutional review board of each medical institution (a list of the institutional review boards has been published elsewhere^28^). The study was conducted in accordance with the principles of the Declaration of Helsinki and Good Clinical Practice guidelines. All patients provided written informed consent before participating in the study. The study was registered at www.clinicaltrials.gov (NCT01855919).

### Study Cohort

Male and female outpatients were eligible for the study if they had CLBP, as defined by Japanese^1^ and international^24^ guidelines, persisting for at least 6 months. Additional inclusion criteria were age 20 to <80 years; used NSAIDs for ≥14 days per month for an average of 3 months before the start of the study and for ≥14 days during the 1-month period before the start of the study (regardless of the dose of NSAIDs and route of administration); and BPI pain severity (average pain) score^29^ of ≥4 at visit 1 (week −1 to −2) and visit 2 (week 0). Patients were excluded if they had radiculopathy or radicular syndrome, a specific disease in the lower back (eg, tumor and myelitis), spinal canal stenosis with neural claudication, hernia with radicular syndrome, or radiating pain in the dermatome in the lower limbs. Other exclusion criteria were history of low back surgery; invasive treatment for the relief of LBP within 1 month before visit 1; requiring crutches or a walker; and having major depressive disorders according to the Mini-International Neuropsychiatric Interview^30^ or suicidal tendencies according to the Columbia-Suicide Severity Rating Scale.^31^

### Treatment Protocol

The study period consisted of a 1- to 2-week pretreatment period, a 14-week treatment period, a 1-week taper period, and a 1-week follow-up period. Patients were randomized to receive duloxetine 60 mg once daily (a 20-mg capsule for 1 week, two 20-mg capsules for 1 week, and three 20-mg capsules for 12 weeks) or matching placebo capsules. The use of drugs with an analgesic effect was permitted as rescue medication for up to 3 consecutive days and for up to a cumulative total of 20 days.

### Assessments

Pain was assessed using the self-administered BPI average pain severity score, which measures average pain during the past 24 hours.^29^ BPI average pain severity scores were rated on a scale ranging from 0 (“no pain”) to 10 (“pain as bad as you can imagine”). LBP-specific quality of life was assessed using the self-administered RDQ-24.^25-27^ For the baseline RDQ-24 score, each item was rated 1 (yes) or 0 (no); the RDQ-24 total score can therefore vary from 0 (no disability) to 24 (maximum disability). For the change in the RDQ-24 score, each item was designated −1 (yes at baseline/no at week 14; improvement), 0 (yes at baseline/yes at week 14 or no at baseline/no at week 14; no change), or 1 (no at baseline/yes at week 14; worsening).

### Efficacy Outcomes

The efficacy outcomes in this post hoc analysis were the change from baseline to week 14 in the BPI average pain severity score and RDQ-24 total score and individual RDQ item scores.

### Statistical Analysis

The post hoc analysis was performed on the full analysis set, which consisted of all randomized patients who received at least one dose of study drug and for whom postbaseline BPI average pain severity scores were available. Analysis of covariance was used to compare the change from baseline to week 14 in the RDQ-24 total score and RDQ items for duloxetine versus placebo, at a significance level of 5%. As this was a post hoc exploratory analysis, no multiplicity adjustment was conducted. Last observation carried forward was used when data at week 14 were missing. Spearman rank correlation coefficients (r) were calculated for (1) change in pain severity (as assessed by the BPI) and change in HRQoL (as assessed by the RDQ items) and (2) change in pain severity and the RDQ item baseline score in duloxetine-treated patients. For the relationship between change in pain severity and the RDQ item baseline score, multiple regression analysis (stepwise forward selection method) was also conducted to identify which baseline RDQ items were predictors of BPI change, at a significance level of 20%. Analyses were performed using SAS version 9.4 (SAS Institute, Cary, NC).

## Results

### Demographic and Baseline Clinical Characteristics

In total, 456 patients were enrolled in the study, of whom 230 patients received duloxetine and 226 patients received placebo. Baseline characteristics were generally similar between the two treatment groups (Table 1). The mean (SD) baseline BPI average score was 5.1 (1.1) and 5.1 (1.0) in the duloxetine and placebo groups, respectively (Table 1). The mean (SD) baseline RDQ-24 total score was 7.59 (4.38) and 7.77 (4.77) in the duloxetine and placebo groups, respectively (Table 1). The two highest-rated individual RDQ items (where higher score = worse status) in both the duloxetine and placebo groups (Table 1) were RDQ02 (“I change position frequently to try and make my back comfortable”; mean [SD]: duloxetine: 0.80 [0.40]; placebo: 0.77 [0.42]) and RDQ13 (“My back hurts most of the time”; mean [SD]: duloxetine: 0.74 [0.44]; placebo: 0.72 [0.45]).

**Table 1:** Patient Demographics and Baseline Characteristics

Parameter^a^	Duloxetine (n = 230)	Placebo (n = 226)	Total (n = 456)
Age, yr	60.0 (13.2)	57.8 (13.7)	58.9 (13.4)
Male, n (%)	115 (50.0)	104 (46.0)	219 (48.0)
Duration of CLBP, yr	9.8 (10.1)	10.3 (10.6)	10.0 (10.3)
BPI average pain score	5.1 (1.1)	5.1 (1.0)	5.1 (1.1)
RDQ-24 total score	7.59 (4.38)	7.77 (4.77)	7.68 (4.57)
RDQ01: I stay at home most of the time because of the pain in my back	0.19 (0.39)	0.22 (0.42)	0.21 (0.40)
RDQ02: I change position frequently to try and make my back comfortable	0.80 (0.40)	0.77 (0.42)	0.79 (0.41)
RDQ03: I walk more slowly than usual because of the pain in my back	0.52 (0.50)	0.54 (0.50)	0.53 (0.50)
RDQ04: Because of the pain in my back, I am not doing any of the jobs that I usually do around the house	0.10 (0.30)	0.07 (0.25)	0.08 (0.28)
RDQ05: Because of the pain in my back, I use a handrail to get upstairs	0.42 (0.49)	0.46 (0.50)	0.44 (0.50)
RDQ06: Because of the pain in my back, I lie down to rest more often	0.40 (0.49)	0.35 (0.48)	0.37 (0.48)
RDQ07: Because of the pain in my back, I have to hold on to something to get out of a reclining chair	0.23 (0.42)	0.31 (0.46)	0.27 (0.44)
RDQ08: Because of the pain in my back, I ask other people to do things for me	0.27 (0.44)	0.31 (0.46)	0.29 (0.45)
RDQ09: I get dressed more slowly than usual because of the pain in my back	0.19 (0.39)	0.22 (0.41)	0.20 (0.40)
RDQ10: I only stand up for short periods of time because of the pain in my back	0.33 (0.47)	0.30 (0.46)	0.32 (0.47)
RDQ11: Because of the pain in my back, I try not to bend or kneel down	0.39 (0.49)	0.43 (0.50)	0.41 (0.49)
RDQ12: I find it difficult to get out of a chair because of the pain in my back	0.12 (0.33)	0.23 (0.42)	0.17 (0.38)
RDQ13: My back hurts most of the time	0.74 (0.44)	0.72 (0.45)	0.73 (0.45)
RDQ14: I find it difficult to turn over in bed because of the pain in my back	0.45 (0.50)	0.44 (0.50)	0.45 (0.50)
RDQ15: My appetite is not very good because of the pain in my back	0.03 (0.16)	0.04 (0.19)	0.03 (0.17)
RDQ16: I have trouble putting on my socks (or stockings) because of the pain in my back	0.43 (0.50)	0.44 (0.50)	0.43 (0.50)
RDQ17: I only walk short distances because of my back pain	0.40 (0.49)	0.34 (0.47)	0.37 (0.48)
RDQ18: I sleep less because of the pain in my back	0.14 (0.35)	0.19 (0.40)	0.17 (0.37)
RDQ19: Because of the pain in my back, I get dressed with help from someone else	0 (0.07)	0 (0.07)	0 (0.07)
RDQ20: I sit down for most of the day because of the pain in my back	0.19 (0.39)	0.17 (0.37)	0.18 (0.38)
RDQ21: I avoid heavy jobs around the house because of the pain in my back	0.54 (0.50)	0.53 (0.50)	0.54 (0.50)
RDQ22: Because of the pain in my back, I am more irritable and bad tempered with people	0.10 (0.30)	0.08 (0.28)	0.09 (0.29)
RDQ23: Because of the pain in my back, I go upstairs more slowly than usual	0.56 (0.50)	0.58 (0.50)	0.57 (0.50)
RDQ24: I stay in bed most of the time because of the pain in my back	0.05 (0.22)	0.06 (0.24)	0.06 (0.23)

BPI = Brief Pain Inventory, CLBP = chronic low back pain, RDQ = Roland-Morris Disability Questionnaire

a Except where otherwise indicated, data are mean (SD).

### Effect of Duloxetine Treatment on the 24-Item Roland-Morris Disability Questionnaire Total Score and Roland-Morris Disability Questionnaire Items

After 14 weeks of treatment, the improvement in the RDQ-24 total score was significantly greater in the duloxetine group than that in the placebo group {least squares (LS) mean difference (95% confidence interval [CI]): −0.64 (−1.25, −0.02); *P* = 0.044} (Table 2). The three individual RDQ items with the greatest improvement for duloxetine versus placebo and which were statistically significant (*P* < 0.05) were RDQ02 (“I change position frequently to try and make my back comfortable”; LS mean difference [95% CI]: −0.17 [−0.26, −0.09]), RDQ17 (“I only walk short distances because of my back pain”; LS mean difference [95% CI]: −0.09 [−0.15, −0.02]), and RDQ13 (“My back hurts most of the time”; LS mean difference [95% CI]: −0.08 [−0.17, −0.00]) (Table 2).

**Table 2:** Least Squares Mean Change From Baseline to Week 14 in the RDQ-24 Total Score and RDQ Items in Duloxetine- and Placebo-treated Patients

RDQ Item^a^	Duloxetine (n = 230), LS Mean (SE)	Placebo (n = 226), LS Mean (SE)	LS Mean Difference (95% CI)	*P*
RDQ-24 total score	−3.86 (0.22)	−3.23 (0.22)	−0.64 (−1.25, −0.02)	0.044
RDQ02	−0.38 (0.03)	−0.21 (0.03)	−0.17 (−0.26, −0.09)	<0.001
RDQ17	−0.19 (0.02)	−0.11 (0.02)	−0.09 (−0.15, −0.02)	0.008
RDQ13	−0.44 (0.03)	−0.36 (0.03)	−0.08 (−0.17, −0.00)	0.047
RDQ03	−0.27 (0.03)	−0.22 (0.03)	−0.05 (−0.13, 0.03)	0.190
RDQ05	−0.20 (0.03)	−0.15 (0.03)	−0.05 (−0.12, 0.02)	0.168
RDQ12	−0.11 (0.02)	−0.07 (0.02)	−0.04 (−0.09, 0.00)	0.066
RDQ16	−0.22 (0.02)	−0.18 (0.02)	−0.04 (−0.11, 0.03)	0.248
RDQ21	−0.20 (0.03)	−0.16 (0.03)	−0.04 (−0.11, 0.04)	0.383
RDQ07	−0.14 (0.02)	−0.11 (0.02)	−0.03 (−0.08, 0.03)	0.382
RDQ23	−0.24 (0.03)	−0.21 (0.03)	−0.03 (−0.11, 0.05)	0.445
RDQ06	−0.21 (0.02)	−0.19 (0.02)	−0.02 (−0.09, 0.04)	0.516
RDQ09	−0.14 (0.02)	−0.12 (0.02)	−0.02 (−0.06, 0.02)	0.400
RDQ20	−0.11 (0.02)	−0.09 (0.02)	−0.02 (−0.06, 0.03)	0.508
RDQ22	−0.07 (0.01)	−0.06 (0.01)	−0.02 (−0.05, 0.01)	0.286
RDQ01	−0.13 (0.02)	−0.11 (0.02)	−0.01 (−0.06, 0.03)	0.538
RDQ10	−0.12 (0.02)	−0.11 (0.02)	−0.01 (−0.08, 0.05)	0.686
RDQ14	−0.24 (0.02)	−0.23 (0.03)	−0.01 (−0.08, 0.06)	0.815
RDQ11	−0.16 (0.03)	−0.17 (0.03)	0 (−0.07, 0.08)	0.912
RDQ19	NA^b^	NA^b^	NA^b^	NA^b^
RDQ18	−0.11 (0.02)	−0.10 (0.02)	0 (−0.04, 0.04)	0.940
RDQ04	−0.05 (0.01)	−0.06 (0.01)	0.01 (−0.02, 0.04)	0.434
RDQ08	−0.13 (0.02)	−0.14 (0.02)	0.01 (−0.05, 0.07)	0.710
RDQ15	−0.01 (0.01)	−0.02 (0.01)	0.01 (−0.01, 0.03)	0.384
RDQ24	−0.03 (0.01)	−0.04 (0.01)	0.01 (−0.02, 0.03)	0.554

CI = confidence interval, LS = least squares, NA = not applicable, RDQ = Roland-Morris Disability Questionnaire, SE = standard error

a See Table 1 for a description of each item number.

b As the value was 0 for all but two patients, the estimators could not be calculated.

### Correlations Between Brief Pain Inventory Change From Baseline and Roland-Morris Disability Questionnaire Item Change From Baseline in Duloxetine-treated Patients

The three highest correlations between BPI change from baseline and RDQ item change from baseline were for RDQ13 (“My back hurts most of the time”; r = 0.344; *P* < 0.001), RDQ23 (“Because of the pain in my back, I go upstairs more slowly than usual”; r = 0.298; *P* < 0.001), and RDQ10 (“I only stand up for short periods of time because of the pain in my back”; r = 0.271; *P* < 0.001) (Table 3).

**Table 3:** Correlation Between BPI Change From Baseline and RDQ Item Change From Baseline in Duloxetine-treated Patients

RDQ Item^a^	Spearman Correlation Between BPI Change From Baseline and RDQ Change From Baseline	*P*
RDQ13	0.344	<0.001
RDQ23	0.298	<0.001
RDQ10	0.271	<0.001
RDQ14	0.226	<0.001
RDQ11	0.223	<0.001
RDQ03	0.206	0.002
RDQ20	0.186	0.005
RDQ21	0.183	0.005
RDQ02	0.181	0.006
RDQ01	0.169	0.010
RDQ08	0.164	0.012
RDQ18	0.154	0.020
RDQ05	0.153	0.020
RDQ16	0.149	0.024
RDQ09	0.117	0.076
RDQ24	0.115	0.081
RDQ12	0.114	0.084
RDQ07	0.113	0.086
RDQ06	0.100	0.130
RDQ04	0.091	0.167
RDQ22	0.074	0.263
RDQ17	0.067	0.313
RDQ15	−0.012	0.860
RDQ19	−0.017	0.801

BPI = Brief Pain Inventory, RDQ = Roland-Morris Disability Questionnaire

a See Table 1 for a description of each item number.

### Correlations Between Brief Pain Inventory Change From Baseline and the Roland-Morris Disability Questionnaire Item Baseline Score in Duloxetine-treated Patients

The correlation coefficient was negative for half of the RDQ items, meaning that higher baseline severity for these RDQ items was correlated with an improvement in pain severity. The three highest correlations between BPI change from baseline and the RDQ item baseline score were for RDQ10 (“I only stand up for short periods of time because of the pain in my back”; r = −0.144; *P* = 0.029), RDQ14 (“I find it difficult to turn over in bed because of the pain in my back”; r = −0.109; *P* = 0.100), and RDQ11 (“Because of the pain in my back, I try not to bend or kneel down”; r = −0.106; *P* = 0.107) (Table 4).

**Table 4:** Correlation Between BPI Change From Baseline and the RDQ Item Baseline Score in Duloxetine-treated Patients

RDQ Item^a^	Spearman Correlation Between BPI Change From Baseline and the RDQ Baseline Score	*P*
RDQ10	−0.144	0.029
RDQ14	−0.109	0.100
RDQ11	−0.106	0.107
RDQ23	−0.091	0.168
RDQ05	−0.061	0.359
RDQ18	−0.060	0.367
RDQ20	−0.054	0.418
RDQ13	−0.029	0.658
RDQ21	−0.023	0.732
RDQ01	−0.014	0.828
RDQ12	−0.013	0.849
RDQ17	−0.001	0.983
RDQ22	0.002	0.972
RDQ07	0.007	0.917
RDQ09	0.011	0.873
RDQ04	0.012	0.857
RDQ19	0.017	0.801
RDQ02	0.018	0.790
RDQ06	0.041	0.537
RDQ08	0.050	0.449
RDQ16	0.051	0.437
RDQ03	0.062	0.346
RDQ24	0.071	0.286
RDQ15	0.090	0.173

BPI = Brief Pain Inventory, RDQ = Roland-Morris Disability Questionnaire

a See Table 1 for a description of each item number.

### Multiple Regression Analysis of Baseline Roland-Morris Disability Questionnaire Items as Predictors of Brief Pain Inventory Change

Multiple regression analysis identified 5 of 24 baseline RDQ items as predictors of BPI change. Multiple regression analysis showed that patients with lower disability as indicated by lower RDQ03 and RDQ06 scores at baseline, and patients with higher disability as indicated by higher RDQ10, RDQ14, and RDQ23 scores at baseline, tended to show a greater improvement in the BPI (Table 5).

**Table 5:** Multiple Regression Analysis

RDQ Item^a^	Correlation Coefficient	*P*^b^
RDQ03	0.540	0.027
RDQ06	0.318	0.156
RDQ14	−0.424	0.045
RDQ23	−0.425	0.080
RDQ10	−0.547	0.018

RDQ = Roland-Morris Disability Questionnaire

a See Table 1 for a description of each item number.

b The significance level was set at 0.20.

### Correlations Between Brief Pain Inventory Change From Baseline and (1) Roland-Morris Disability Questionnaire Item Change From Baseline and (2) the Roland-Morris Disability Questionnaire Item Baseline Score in Duloxetine-treated Patients

The 24 RDQ items were categorized into four subgroups according to the two types of correlations evaluated in duloxetine-treated patients: (1) correlations between BPI change from baseline and RDQ item change from baseline and (2) correlations between BPI change from baseline and the RDQ item baseline score (creating the 2 × 2 table shown in Table 6). For 14 of the 24 RDQ items, the correlation between BPI change from baseline and RDQ item change from baseline was statistically significant (Table 6). For 4 of these 14 items, the correlations between BPI change from baseline and the RDQ item baseline score were positive: RDQ02, RDQ03, RDQ08, and RDQ16 (Table 6). For 10 of these 14 items, the correlations between BPI change from baseline and the RDQ baseline score were negative: RDQ01, RDQ05, RDQ10, RDQ11, RDQ13, RDQ14, RDQ18, RDQ20, RDQ21, and RDQ23 (Table 6).

**Table 6:** Correlations Between BPI Change From Baseline and (1) RDQ Item^a^ Change From Baseline and (2) the RDQ Item Baseline Score

		Correlation Between BPI Change From Baseline and RDQ Item Change From Baseline	
		Yes (Cutoff: *P* < 0.05)	No (Cutoff: *P* ≥ 0.05)
Correlation between BPI change from baseline and the RDQ item baseline score	Positive correlation (r > 0)	RDQ02, RDQ03,^b^ RDQ08, and RDQ16	RDQ04, RDQ06,^b^ RDQ07, RDQ09, RDQ15, RDQ19, RDQ22, and RDQ24
	Negative correlation (r < 0)	RDQ01, RDQ05, RDQ10,^b^ RDQ11, RDQ13, RDQ14,^b^ RDQ18, RDQ20, RDQ21, and RDQ23^b^	RDQ12 and RDQ17

BPI = Brief Pain Inventory, RDQ = Roland-Morris Disability Questionnaire

a See Table 1 for a description of each item number.

b Identified by multiple regression analysis (Table 5).

## Discussion

Treatment goals for patients with CLBP are to improve HRQoL and to reduce pain intensity, while avoiding potential negative effects of treatment. For example, the addictive nature of opioids has led to serious opioid-related epidemics, with opioid use being associated with long-term disability,^32^ while not demonstrating any advantage over the use of nonopioid medications with respect to improving pain-related function.^33^ Although comprehensive and multidisciplinary interventions are necessary, especially in severe cases of CLBP, pharmaceutical intervention is one of the key components of CLBP treatment.^34^ Therefore, it would be of practical value for physicians to know which aspects of HRQoL could be improved with pharmaceutical interventions to guide treatment decisions and inform patients of likely treatment outcomes. It would also be clinically relevant if physicians are able to anticipate which patient cohorts, in the context of the RDQ-24 profiles, respond more substantially to an analgesic, leading to a higher level of pain reduction. Although the chronic pathophysiology is different from that of CLBP, previous studies in patients with diabetic painful neuropathy (DPNP) found correlations between improvement in the pain score and improvement in HRQoL in duloxetine-treated patients with DPNP.^35^ In addition, another study of DPNP showed different pain improvement profiles in terms of the Neuropathic Pain Symptom Inventory between duloxetine and pregabalin.^36^ However, there is limited literature addressing the qualitative assessment of HRQoL as a predictor of treatment outcomes or responder profiles in the treatment of CLBP. In this study of Japanese patients with CLBP in which duloxetine improved both pain severity and HRQoL (as assessed by the RDQ-24 total score), the current post hoc analysis created a profile of the 24 individual RDQ items by categorizing them into four subgroups based on the following two types of correlations in duloxetine-treated patients: (1) correlations between BPI change from baseline and RDQ item change from baseline and (2) correlations between BPI change from baseline and the RDQ item baseline score. As far as we know, our study is the first report of such an analysis in patients with CLBP.

As reported previously,^19^ duloxetine treatment significantly improved the RDQ-24 total score compared with placebo in this cohort of patients with CLBP. The current post hoc analysis extends these findings by showing different degrees of improvement in the individual 24 RDQ items by duloxetine treatment in this study cohort. In particular, the RDQ02, RDQ13, and RDQ17 items were statistically significantly improved by duloxetine treatment compared with placebo. As the RDQ02 and RDQ13 items were the two worst-rated items at baseline (mean of 0.79 and 0.73, respectively, in the overall study cohort), this suggests that duloxetine has a clinically relevant treatment effect on HRQoL that had deteriorated because of CLBP.

Irrespective of treatment differences relative to placebo, correlations between BPI change from baseline and RDQ item change from baseline in this post hoc analysis varied in strength for the 24 RDQ items. The strongest correlations between BPI change from baseline and RDQ item change from baseline were RDQ13, RDQ23, and RDQ10, suggesting that duloxetine-treated patients with CLBP who have improvements in these three RDQ items may have a better pain response. Of note, for 14 of the 24 RDQ items (58.3%), the correlations were statistically significant (*P* < 0.05), which implies that pain reduction is clinically important in improving disease-specific HRQoL in CLBP. Correlations between improvement in the pain score and improvement in HRQoL have also been reported for duloxetine-treated patients with DPNP.^35^ In a post hoc analysis of pooled data from three randomized controlled trials of duloxetine in patients with DPNP (335 duloxetine-treated patients), improvements in 24-hour average pain severity scores were correlated with improvements in HRQoL as assessed by the 36-Item Short-Form Health Survey subscales (Pearson correlation coefficients ranging from 0.114 to 0.401) and the European Quality of Life 5-Dimensions questionnaire (Pearson correlation coefficient of 0.271).

Despite its overall efficacy as an analgesic, duloxetine has been reported to be more effective in some patient cohorts than others.^9,28^ CLBP pathophysiology has multiple aspects, including nociceptive, neuropathic, socioeconomical, and psychogenic factors, and, therefore, patient cohorts with CLBP are heterogeneous. The RDQ-24 includes questions related to these pathophysiological elements. Thus, in the current post hoc analysis, we investigated which baseline characteristics in terms of the RDQ-24 were associated with a more substantial response to duloxetine. Assessment of correlations between BPI change from baseline and RDQ item baseline severity showed both negative (12 items) and positive (12 items) correlations in this cohort of Japanese patients with CLBP. Although the size of the correlation coefficient was generally small, the RDQ items with negative correlations at baseline might be candidates as predictors of the level of pain reduction occurring after treatment. Of note, similar results were obtained from the correlation and multiple regression analyses: three of the four RDQ items for which higher baseline severity predicted greater pain reduction in the correlation analysis (RDQ10, RDQ14, and RDQ23) were also identified in the multiple regression analysis. These analyses imply that these three RDQ items might be used as baseline predictors to estimate the posttreatment reduction in pain. It remains to be seen whether the classification of the 24 RDQ items into four categories based on correlations between BPI change from baseline and (1) RDQ change from baseline and (2) the RDQ baseline score in duloxetine-treated patients would allow physicians to predict a level of response to duloxetine treatment and its possible outcomes in terms of disease-specific HRQoL.

The use of patient-reported outcome measures to predict response to duloxetine has been shown for patients with DPNP in the COmbination vs. Monotherapy of pregaBalin and dulOxetine in Diabetic Neuropathy study, which assessed high-dose duloxetine or pregabalin monotherapy or duloxetine plus pregabalin combination therapy.^36,37^ Exploratory post hoc analyses of neuropathic pain symptoms using the Neuropathic Pain Symptom Inventory in this randomized phase 3 study identified subgroups of patients with distinct pain characteristics at baseline who had responded differently to duloxetine and pregabalin when given alone or in combination.^36^ The authors of this analysis suggested that taking heterogeneity in the patient cohort into account would allow for a more stratified, or even personalized, treatment approach for patients with chronic pain conditions.^36^

We note that the safety profile for duloxetine in the current study was similar to that reported in studies of patients receiving duloxetine for other pain indications.^19^ As reported previously, patients receiving duloxetine in the current study reported a higher incidence of somnolence (19.2% versus 7.1%), constipation (10.7% versus 2.2%), nausea (9.0% versus 2.7%), dizziness (6.4% versus 0.9%), and dry mouth (6.0% versus 0%) compared with patients receiving placebo.^19^ Most of these adverse events were mild or moderate in severity and were resolved or improved.^19^

This was the first analysis of the relationship between pain and HRQoL in duloxetine-treated patients with CLBP. Strengths of this analysis included analyzing data from a randomized, placebo-controlled, double-blind, phase 3 study and the use of an HRQoL measure specific for back pain. Limitations included the post hoc nature of the analysis and the possible difficulty of applying the results of this analysis to clinical practice. As the study was conducted in Japanese patients alone and the duration of duloxetine treatment in the study was relatively short, it might be difficult to generalize the results of the analysis to other cohorts and to longer periods of treatment. In addition, as each RDQ item in the RDQ-24 has only two categories (yes/no), it might be difficult to detect small changes in HRQoL. However, this may also be a strength as it means that changes in HRQoL would need to be clinically relevant and detectable by the patient as well as the physician and, therefore, less likely to be an artifact. In addition, some RDQ items had low proportions of patients responding “Yes” at baseline, making it difficult to evaluate correlations between RDQ baseline severity and pain reduction.

In conclusion, this post hoc analysis suggested that improvement in pain severity was associated with improvement in aspects of disease-specific HRQoL in duloxetine-treated patients with CLBP. In particular, positive responses to duloxetine in terms of the RDQ13, RDQ23, and RDQ10 items may correlate with better pain responses. However, further investigation is required to fully elucidate the relationship between pain and HRQoL in duloxetine-treated patients with CLBP. Nevertheless, given the difficulty in treating CLBP and the ease of administering HRQoL measures such as the RDQ-24 in daily clinical practice, determining which baseline HRQoL characteristics might be predictors of response to duloxetine would provide valuable information for making treatment decisions and anticipating treatment outcomes in terms of HRQoL in patients with CLBP.
